# The Assessment of Structural Validity and Internal Consistency of the Polish Health Literacy Questionnaire (HLQ) in Primary Care Settings

**DOI:** 10.3390/healthcare14193320

**Published:** 2026-10-06

**Authors:** Agnieszka Lipiak, Barbara Gawłowska, Marlena Szewczyczak, Monika Karasiewicz, Rafał Staszewski, Richard Osborne, Melanie Hawkins, Ewelina Chawłowska

**Affiliations:** 1 Department of Preventive Medicine, Poznan University of Medical Sciences, 60-781 Poznan, Poland; 2 Global Health and Equity Development Hub, La Trobe Rural Health School, La Trobe University, Bendigo, VIC 3550, Australia

**Keywords:** Health Literacy Questionnaire (HLQ), health literacy, health literacy measurement, health literacy development, psychometrics, validation, validity testing, primary health care, patients, Poland

## Abstract

Background/Objectives: Health literacy extends beyond individual competencies and encompasses how people engage with health systems. The Health Literacy Questionnaire (HLQ) is a widely used multidimensional instrument designed to assess different health literacy aspects across nine distinct domains. This study aimed to examine structural validity and internal consistency of the Polish version of the HLQ among primary care service users. Methods: The HLQ was used in a cross-sectional survey among 360 adults (mean age 51.76 years, SD 17.48) attending four primary care clinics in Poland. The assessment included confirmatory factor analysis (CFA) and internal consistency tests. Results: A nine-factor CFA model was applied to the HLQ items, without allowing cross-loadings or correlated residuals. The fit indices confirmed good model fit: χ^2^_WLSMV_ (df = 866) = 1175.63, *p* < 0.001; CFI = 0.98; TLI = 0.98; RMSEA = 0.038; and SRMR = 0.056. The factor loadings ranged from 0.55 to 0.94, indicating acceptable item–factor relationships across all domains. Internal consistency was satisfactory across all nine domains, with composite reliability ranging from 0.76 to 0.87 and Cronbach’s alpha from 0.78 to 0.88. Conclusions: The findings provide preliminary evidence supporting structural validity and internal consistency of the Polish HLQ in this sample of primary care service users and suggest the potential value of the instrument for multi-dimensional assessment of health literacy in similar Polish primary care populations. Future studies should evaluate additional measurement properties of the tool, as well as the performance of the instrument in nationally representative samples and across diverse clinical settings.

## 1. Introduction

People’s health and well-being are fundamentally influenced by the effective use of health information and health services. This practice is captured by the concept of health literacy, defined by the World Health Organization (WHO) as “people’s knowledge, confidence and comfort—which accumulate through daily activities and social interactions and across generations—to access, understand, appraise, remember and use information about health and health care, for the health and well-being of themselves and those around them” [1]. Through this definition, it becomes evident that health literacy is not merely a set of individual actions or competencies but also a dynamic social practice shaped by the communities and settings in which we live and function [2]. Among these, the health system plays a pivotal role, sometimes facilitating and sometimes hindering the ways people engage with health information and services. Health systems vary widely in terms of their health literacy responsiveness, i.e., their capacity to accommodate diverse health literacy needs and create environments that promote equitable access to and engagement with health information and services [1]. Less responsive systems often confront users with overwhelming and contradictory health information, compounded by the difficulty of navigating fragmented and inaccessible health services [3,4]. The resulting healthcare barriers often disproportionately affect groups that experience vulnerability such as older adults, persons with chronic conditions, immigrants, and minorities [5,6,7,8,9,10]. Thus, international frameworks emphasise that it is a core public responsibility to design health literacy-responsive health systems to ensure that no one is left behind [1,11,12]. Achieving this goal requires identifying local health literacy needs and assessing the level of system responsiveness. This should be informed by a context-specific understanding of health literacy and supported by appropriate, locally relevant measurement tools that capture the social aspect of the concept [13].

The available research on health literacy needs in Polish populations has employed several measurement instruments. Some studies utilised scales that were developed in the native language but lacked comprehensive testing [14,15]. One study made use of an instrument that was limited to reading- and writing-related health literacy and did not address its social dimension [16]. Two broader health literacy questionnaires that have been translated and used in adult Polish populations include the European Health Literacy Survey Questionnaire (HLS-EU-Q) [17,18] and Functional, Communicative and Critical Health Literacy scale (FCCHL) [19]. However, the former has no specific focus on social engagement and has been used mainly for cross-sectional surveys and country comparisons [20], while the latter is a relatively short 14-item scale covering three broad domains [21]. For this reason, we decided to translate into Polish a questionnaire that would allow for a more nuanced assessment of both health literacy strengths and challenges to go beyond measuring individual skills and capture the interplay between people and their local health systems.

The Health Literacy Questionnaire (HLQ), developed in Australia [22] and adapted for numerous other communities and settings [23], meets this criterion: it measures nine distinct domains spanning cognitive, social, and system navigation aspects. This multidimensionality allows researchers to pinpoint the specific strengths and challenges people have when engaging with health information within local health systems and within their own social support networks. By 2024, it had been translated into 40 languages and has undergone contemporary psychometric testing in numerous studies across diverse populations, consistently demonstrating strong psychometric properties across countries and languages [23,24]. For example, studies on French [25], Danish [26], Portuguese [27], German [28], Korean [29] and Dutch [30] language versions of the questionnaire supported the original nine-factor structure of the HLQ. Across these studies, confirmatory factor analyses demonstrated at least satisfactory model fit, while internal consistency coefficients measured as Cronbach’s α or composite reliability were generally good to excellent. Several studies provided evidence of satisfactory test–retest reliability [31,32] and known-groups validity [30,33] of HLQ translations.

Taking the above into account, we decided to undertake the linguistic and cultural adaptation of the HLQ for use in the Polish context. Two samples were selected for testing the psychometric properties of the adapted questionnaire: health professions students and primary care service users. The results obtained in the student sample are presented elsewhere [34]. The present paper focuses on the latter group. Primary care service users were chosen because of their active health system engagement, which could be captured by means of the system-related HLQ scales. As the main entry point to the health system, primary care is likely to reach a wide spectrum of the population with varying levels of health literacy, making it a suitable setting for obtaining a diverse sample for validation processes. This paper presents the results of the assessment of structural validity and internal consistency of the linguistically and culturally adapted Polish HLQ conducted in the sample of people attending Polish primary care centres. We hypothesised that the original nine-factor structure of the HLQ would be supported in the Polish primary care sample and that all nine scales would demonstrate satisfactory internal consistency.

## 2. Materials and Methods

### 2.1. Sampling and Data Collection

A cross-sectional study using the HLQ (44 items), questions related to health behaviours (22 items) and additional sociodemographic questions (14 items) was conducted among attendees at four primary care clinics in the city of Poznan, Wielkopolskie province, Poland, from September 2023 to May 2024. The inclusion criteria were being an adult (≥18 years) attending one of the primary care clinics, having the ability to use Polish fluently, and being willing and able to consent to participate.

The survey was conducted after the Bioethics Committee of the Poznan University of Medical Sciences confirmed that the study did not qualify as a scientific experiment and therefore a waiver of further ethical review of the protocol was granted (decision no. KB—465/23 of 24 May 2023).

All the participating clinics consented to surveying of their service users. Convenience sampling was used. Based on previous simulation research, a sample size of at least 300 was considered likely to provide adequate conditions for the planned confirmatory factor analysis [35]. The survey was not publicly advertised. Participants were recruited opportunistically through a single in-person contact: they were either approached in waiting rooms of the clinics or reached in their homes during routine home visits by primary care nurses. The participants were informed about the purpose of the survey, its fully anonymous nature, and their right to participate voluntarily or withdraw from the survey at any time. No incentives were offered for participation. After providing written consent to participate, the participants were asked to complete an in-person, pen-and-paper questionnaire. The interviewers took care to ensure privacy of the process and assisted the respondents with reading out items when necessary. The questionnaire consisted of 5 pages, with 12 to 22 items per page, and typically took the respondents 10–15 min to complete. The recruitment process resulted in a final sample of 360 adult primary care service users.

### 2.2. The Tool

The original Health Literacy Questionnaire, developed in 2013 using a validity-driven systematic grounded approach [22], consists of 44 items in nine domains (scales):

1. Feeling understood and supported by healthcare providers
2. Having sufficient information to manage my health
3. Actively managing my health
4. Social support for health
5. Appraisal of health information
6. Ability to actively engage with healthcare providers
7. Navigating the healthcare system
8. Ability to find good health information
9. Understand health information well enough to know what to do

The first part of the HLQ (domains 1–5) employs a 4-point Likert-type scale, where respondents indicate their level of agreement with statements ranging from “strongly disagree” to “strongly agree”. The second part (domains 6–9) uses a 5-point Likert-type scale to assess task difficulty, with response options ranging from “cannot do or always difficult” to “always easy”. There is no overall HLQ score; the nine domains are scored separately. The domain scores are calculated as the mean of item responses within each domain.

A licence to use and translate the HLQ (licence no. TL1902IA) had been obtained from the authors, who required that the Polish translation and adaptation adhere to the Translation Integrity Procedure [36]. The procedure emphasised the importance of achieving construct equivalence and clear, natural language in the translation. The questionnaire was forward-translated by a professional translator and subsequently reviewed by a second professional translator. Translation decisions were informed by the HLQ item intent descriptions provided by the questionnaire developers. Discrepancies and alternative wording options were discussed during three consensus meetings involving both translators and a public health researcher fluent in English. To resolve remaining uncertainties, selected item variants were piloted with community members representing potential future respondents. A provisional consensus Polish version was then independently back-translated into English by a native English speaker fluent in Polish. The consensus forward translation and the back translation, together with the translators’ comments, were reviewed by the original HLQ developers, whose feedback informed the preparation of the final pre-test version. It was used in cognitive interviews with twelve participants: six primary care service users and six university students. These two groups were selected because they represented the two populations included in the broader validation study of the Polish HLQ. The former group—primary care service users—were purposively selected to ensure variation in age, sex, educational attainment, and occupational background. They ranged in age from 30 to 82 years and represented vocational, secondary, and higher education levels. Occupational backgrounds included blue-collar and white-collar workers, retired individuals, and a rural worker. This diversity was intended to support evaluation of the comprehensibility and cultural appropriateness of the Polish HLQ items across individuals with different social and educational backgrounds.

Interviews were conducted face to face in accordance with a cognitive interviewing guide, also provided by the authors of the original HLQ. After independent questionnaire completion, participants were asked to explain in their own words what each item meant and why they selected particular responses. Special attention was paid to signs that an item was difficult for a participant to interpret or respond to. Participants’ interpretations of the items were compared with the original item intent descriptions. Thus, the interviews enabled the assessment of comprehension and conceptual equivalence of the original and adapted items. All interviewees demonstrated an understanding of the items that was consistent with their intended meaning. As a result, the items were retained as originally formulated, followed by validity testing in a sample of people attending primary care.

### 2.3. Statistical Analysis

PQStat v. 1.8.6 (PQStat Software, Poznan, Poland) was used for descriptive analyses.

To characterise response patterns, dichotomised proportions of responses indicating challenge were calculated for both individual items and for each of the nine scales. The values represented the percentage of respondents selecting stronger response categories. In the first part of the HLQ (scales 1–5), responses were dichotomised into “strongly disagree/disagree” versus “agree/strongly agree”. In the second part (scales 6–9), responses were dichotomised into “cannot do or always difficult”, “usually difficult” and “sometimes difficult” versus “usually easy” and “always easy”. These values, used in a number of previous studies on HLQ adaptations and sometimes reported as difficulty levels or difficulty estimates, served as a quick overview of response patterns across items and scales and did not represent psychometric item-difficulty parameters [25,26,27,37].

Further analyses included internal consistency testing for each HLQ scale (Cronbach’s α and composite reliability). Composite reliability (CR) was estimated using a model-based approach. Ninety-five percent confidence intervals (95% CI) for CR were obtained using a nonparametric bootstrap procedure with 2000 resamples. Next, confirmatory factor analyses (CFA) were conducted using R Studio version 2024.04.2.764 (Posit Software, PBC, Boston, MA, USA) with the lavaan and semTools packages (version 0.6.18) [38,39]. Consistent with the original HLQ measurement model, a nine-factor structure was specified a priori. A restrictive nine-factor model was estimated without allowing cross-loadings or correlated residuals. The prespecified model was evaluated without introducing post hoc modifications based on statistical fit indices.

The response options were treated as ordinal variables (coded 1–4 for disagree/agree items and 1–5 for difficult/easy items). Model estimation was performed using the weighted least squares mean and variance adjusted (WLSMV) method and the robust/scaled values. Model fit was evaluated using several indices, including χ^2^, the comparative fit index (CFI), Tucker–Lewis index (TLI), standardised root mean residual (SRMR), and root mean square error of approximation (RMSEA). A model was considered to demonstrate good fit if it met the following criteria: CFI > 0.95, TLI > 0.95, RMSEA < 0.06, and SRMR ≤ 0.08 [40]. To assess the adequacy of item–factor relationships, standardised factor loadings were inspected, with 0.50 adopted as the minimum acceptable value [41]. The latent factors were identified using the fixed-factor variance method.

Prior to analysis, the dataset was screened for completeness and consistency of responses. Questionnaires with more than 50% of the HLQ items missing were excluded from analysis. Missing data were infrequent and were distributed across individual items. Missing HLQ item responses were imputed using the Expectation-Maximisation algorithm, provided that no more than two items were absent in 4- or 5-item scales and no more than three items were absent in 6-item scales [42].

## 3. Results

### 3.1. Sample Characteristics

The study sample comprised 360 primary care users. The participants were aged 19–90 years, with a mean of 51.76 years (SD = 17.48). A majority (93.89%) were native Polish speakers, women (62.50%), large city residents (64.72%), and persons with at least one physician-diagnosed chronic condition (63.61%). Detailed sample characteristics are presented in Table 1. All variables were self-reported by participants.

### 3.2. Proportions of Responses Indicating Challenge

The proportions of responses indicating challenge for individual items and particular scales were assessed. The highest proportions of respondents selecting stronger responses were found in scales 6. Ability to actively engage with healthcare providers (mean 54.94%) and 8. Ability to find good health information (mean 51.40%). The lowest proportions were observed in scales 1. Feeling understood and supported by healthcare providers (mean 20.98%) and 4. Social support for health (mean 28.09%). The proportions varied substantially across items within most scales, with ranges spanning from 13.25 (Scale 3. Actively managing my health) to 37.99 (Scale 4. Social support for health) in Part 1 of the questionnaire, and from 18.61 (Scale 8. Ability to find good health information) to 31.31 (Scale 6. Ability to actively engage with healthcare providers) in Part 2. Details are presented in Table 2.

### 3.3. Internal Consistency of the Nine HLQ Factors

Internal consistency of the nine scales was assessed using Cronbach’s alpha (α) and composite reliability (CR) [43]. The scale with the lowest reliability was 5. Appraisal of health information (CR = 0.76), although this value was still above the preferred threshold (≥0.70). All other scales were approximately between 0.8 and 0.9. None of the scales had a reliability coefficient ≥0.95, which is sometimes used as a threshold for flagging potential item redundancy or an excessively narrow construct (Table 3).

### 3.4. Confirmatory Factor Analysis

To assess model fit, confirmatory factor analysis (CFA) was conducted. A nine-factor CFA model was applied to the HLQ items, without allowing cross-loadings or correlated residuals. The fit indices confirmed good model fit: χ^2^_WLSMV_ (df = 866) = 1175.63, *p* < 0.001; CFI = 0.98; TLI = 0.98; RMSEA = 0.038; and SRMR = 0.056. The factor loadings for all items were above the acceptable threshold of 0.50 (Table 4), ranging from 0.55 to 0.94. Confidence intervals for the factor loadings are presented in Table S1 (Supplementary Materials).

### 3.5. Factor Correlations

Table 5 presents correlations between the nine HLQ factors (scales). The strongest correlations were found in Part 2 of the HLQ: between scales 6. Ability to actively engage with healthcare providers and 7. Navigating the healthcare system (0.93), as well as between scales 8. Ability to find good health information and 9. Understand health information well enough to know what to do (0.89). With the exception of one more value (0.81), all other correlations were below 0.80. In contrast, the lowest correlation was found between scales 1. Feeling understood and supported by healthcare providers and 3. Actively managing my health (0.26).

To further examine the relationships between the scales, discriminant validity was additionally evaluated using the heterotrait–monotrait ratio of correlations (HTMT). The highest HTMT values were observed for scales 6 and 7 (0.93) and scales 8 and 9 (0.89). HTMT values for all HLQ scale pairs are reported in Table S2 (Supplementary Materials).

## 4. Discussion

This study presents an examination of selected psychometric properties of the Polish version of HLQ in primary care settings. The linguistic and cultural adaptation of the instrument was guided by the strict Translation Integrity Procedure [36]. The psychometric analyses applied to the HLQ were grounded in contemporary classical test theory and used in a number of validity-testing studies on different language versions of the HLQ [25,26,37]. The analyses comprised a description of response distribution, internal consistency tests, confirmatory factor analysis, and factor correlations. The findings provide evidence supporting the structural validity and internal consistency of the Polish HLQ in this sample of primary care service users.

The descriptive analysis showed substantial variation in proportions of responses indicating challenge across items of Polish HLQ: some items were relatively easy for respondents to endorse positively, while for other items, the strongest response categories were selected less frequently. The largest intra-scale range of values (min 10.89, max 48.88, range 37.99) was recorded in scale 4. Social support for health. The smallest, but still significant, variation was found in scale 3. Actively managing my health (min 22.5, max 35.8, range 13.3). This variability is consistent with the rationale underlying the original HLQ, in which items were intentionally designed to capture a broad range of experiences and challenges within each health literacy domain [22]. Substantial variability was also observed between scales. The highest proportion of responses indicating challenge was 54.94 in Scale 6. Ability to actively engage with healthcare providers. The lowest proportion was found in Scale 1. Feeling understood and supported by healthcare providers (20.98). These differences suggest the potential for the Polish HLQ to capture distinct health literacy profiles across different domains. An important strength of this profile-based approach is that it preserves the multidimensional nature of health literacy, unlike instruments that summarise it in a single overall score [44]. It should be noted, however, that our analysis involved dichotomisation of the original ordinal response categories. Consequently, the findings should be interpreted as reflecting response distributions rather than the full spectrum of response intensity.

The next analyses provided evidence of satisfactory internal consistency across all the nine HLQ scales, with Cronbach’s α values from 0.78 to 0.88 and CR from 0.76 to 0.87. Comparable CR ranges were recorded in validity-testing studies conducted in geographically close countries, for instance Denmark (0.77–0.87) [26] and France (0.77–0.91) [25], while bigger variation was reported in some more distant locations such as Brazil (0.67–0.82) [45] and Ghana (0.66–0.82) [46]. In our study, the lowest internal consistency was found in scales 5 and 9 related to, respectively, the appraisal and understanding of health information. Each of the two scales contained one item with a factor loading below 0.60. Overall, these findings suggest that the Polish HLQ scales were sufficiently homogeneous and may provide reliable scores for group-level comparisons.

Our subsequent analyses encompassed classical CFA with a restrictive approach. We sought to determine if the original a priori nine-scale factor structure proposed in 2013 [22] was reproducible in Polish-speaking adults attending primary care clinics. The prespecified restrictive nine-factor model, in which cross-loadings and correlated residuals were constrained to zero, demonstrated good overall model fit as measured with χ^2^_WLSMV_, CFI, TLI, RMSEA and SRMR indices [40]. In addition, among the 44 items of the Polish HLQ, all factor loadings were above the minimum threshold of 0.50 acceptable in CFA of multifaceted constructs [41] and ranged from 0.55 to 0.94. Only three items had loadings below 0.60, indicating generally strong relationships between the items and their respective latent factors. Our results are comparable to those observed in studies examining structural validity of HLQ adaptations across different languages. Evidence supporting the replication of the original nine-factor structure was similarly found among persons recruited in Danish primary care settings, public places and workplaces [26], in a German sample of patients with chronic conditions [28], among Norwegian adults with psoriasis [47], in general adult Slovak population [48], and in persons with chronic diseases from Korea [29], among other studies.

Next, we examined the relationships between the HLQ dimensions. Factor correlations among the nine HLQ scales ranged from 0.26 to 0.93. Overall, the pattern of correlations was consistent with the proposed nine-factor structure, although several high correlations, particularly among the Part 2 scales, suggested overlap between some constructs. Whereas the values did not exceed 0.75 between the scales in Part 1 of the questionnaire, they tended to be higher in Part 2 (0.73–0.93). The highest correlations were observed between the factors representing engagement with healthcare providers and system navigation (scales 6 and 7), as well as between finding health information and understanding it (scales 8 and 9). To further examine discriminant validity, HTMT ratios were calculated. Using the conservative threshold of 0.85 [49], discriminant validity was supported for most scale pairs. However, the HTMT values for scales 6 and 7 (0.93) and scales 8 and 9 (0.89) exceeded this criterion, confirming substantial overlap between these constructs. This pattern was first reported by the authors of the original instrument [22] and has since been observed across several language adaptations of the HLQ [25,26,28,29]. It was suggested that the higher correlations in Part 2 may be attributable to the presence of a higher order factor, for example a factor encompassing a more general capability to engage with the healthcare system [50]. Nevertheless, the high correlations and HTMT values found in our study may signal insufficient discriminant validity of the scales in Part 2. Although community research reported different sociodemographic correlates for these scales [24], such findings do not constitute direct evidence of discriminant validity in the present sample.

This work was undertaken in a Polish primary care setting among people with diverse health conditions, including those typical of the 50+ years population, such as hypertension and diabetes. Given the evidence supporting its structural validity and internal consistency in this sample, the HLQ is likely to be a useful tool to measure health literacy diversity among Polish primary care users. The HLQ is intended to provide in-depth and nuanced data across nine dimensions for understanding populations, informing the development of programmes and policies, and evaluating interventions. The data presented in this study support the use of the Polish HLQ for these purposes within primary care settings, although further research is needed before its applicability to other populations and healthcare contexts can be established.

At the same time, these findings should be interpreted in light of several limitations of the study. We used convenience sampling in four clinics located in one urban area. While 18.9% of the participants reported living in villages and 15.6% in towns, these were likely near the big city of Poznan. Women were overrepresented (62.5% vs. 52.1% in Wielkopolskie province adult population) [51], possibly due to women’s greater survey responsiveness and higher primary care use (57.1% of visits in 2023 by female service users) [52]. The participants also had higher education levels than the general population (82.2% with at least secondary education vs. 60.2% reported in this province) [53]. Moreover, our study did not systematically record the numbers of individuals approached, those who declined participation, or those who were excluded. Consequently, a response rate could not be calculated. The absence of these data precludes an assessment of potential non-response bias. The individuals who agreed to participate may have differed systematically from those who did not participate, for example in their interest in health-related issues, willingness to engage in research, or health literacy levels. Sampling in an ambulatory care setting may have further biased the sample toward individuals already engaged with health services. Together, these factors limit the generalisability of the results to the broader Polish adult primary care population. Therefore, the results should be interpreted primarily as evidence of the psychometric performance of the Polish HLQ in the recruited sample rather than as evidence of its performance in the general Polish population. In addition, the study was not preregistered, and the analysis plan was not made publicly available before the analyses were conducted, so the possibility of analytical flexibility cannot be fully excluded. Finally, the study assessed structural validity and internal consistency only. Other measurement properties, including test–retest reliability, measurement invariance, responsiveness and measurement error, were not evaluated and should be examined in future studies. Additional aspects of construct validity assessed through hypothesis testing (e.g., convergent validity, known-groups validity, hypothesised associations with external variables) also remain to be established. Further research is needed to clarify the discriminant validity of the Part 2 scales, for which high inter-factor correlations and HTMT values indicated substantial construct overlap.

Nonetheless, the study fills a gap in research on health literacy of Polish primary care service users by offering a multidimensional measurement tool originally developed with a validity-driven approach and extensively evaluated in previous international research. Additionally, the adaptation process of the Polish HLQ and its subsequent testing adhered to well-established procedures, including restrictive confirmatory factor analyses.

## 5. Conclusions

The Polish HLQ demonstrated evidence supporting its structural validity and internal consistency in this sample of primary care service users. These findings are consistent with the items and constructs being well understood and meaningful to community members. Therefore, the HLQ may be useful for assessing multidimensional health literacy profiles and identifying health literacy needs in similar Polish primary care populations. Further research is needed to establish the performance of the instrument in other population groups, in nationally representative samples, and across diverse healthcare settings.

## Figures and Tables

**Table 1:** Sample characteristics.

*n* = 360		*n* (%)
Sex	Female	225 (62.50%)
	Male	130 (36.11%)
	Missing	5 (1.39%)
Age (years)	<25	21 (5.83%)
	25–34	44 (12.22%)
	35–44	49 (13.61%)
	45–54	69 (19.17%)
	55–64	48 (13.33%)
	65–74	72 (20.00%)
	75≤	31 (8.61%)
	Missing	26 (7.22%)
BMI	Underweight (<18.50)	7 (1.94%)
	Normal (18.50–24.99)	137 (38.06%)
	Overweight (25.00–29.99)	111 (30.83%)
	Obesity (≥30.00)	53 (14.72%)
	Missing	52 (14.44%)
Education	Primary	30 (8.33%)
	Vocational	30 (8.33%)
	Secondary	151 (41.94%)
	Higher	145 (40.28%)
	Missing	4 (1.11%)
Native language	Polish	338 (93.89%)
	Other	5 (1.39%)
	Missing	17 (4.72%)
Residence	Village	68 (18.89%)
	Small town (<50,000 inhabitants)	36 (10.00%)
	Medium-sized town (50,000–200,000 inhabitants)	20 (5.56%)
	Large city (>200,000 inhabitants)	233 (64.72%)
	Missing	3 (0.83%)
Financial situation	Very bad	4 (1.11%)
	Bad	14 (3.89%)
	Average	127 (35.28%)
	Good	153 (42.50%)
	Very good	46 (12.78%)
	Missing	16 (4.44%)
Number of physician-diagnosed chronic conditions	0	131 (36.39%)
	1	107 (29.72%)
	2≤	122 (33.89%)
Hypertension	Yes	128 (35.56%)
	No	232 (64.44%)
Diabetes	Yes	53 (14.72%)
	No	307 (85.28%)

**Table 2:** Mean proportions of responses indicating challenge of HLQ items and scales and mean scale scores in the study sample (*n* = 360).

Part/Scale/Item *	Proportions of Responses Indicating Challenge ** (%)	Scale Scores *** (M ± SD)
Part 1		(scored on a 1–4 scale)
1. Feeling understood and supported by healthcare providers	20.98	2.87 ± 0.65
I have at least one healthcare provider who…	25.63	
I have at least one healthcare provider I can…	29.53	
I have the healthcare providers I need…	10.34	
I can rely on at least one…	18.44	
Range ****	19.19	
2. Having sufficient information to manage my health	34.92	2.67 ± 0.60
I feel I have good information about health…	36.59	
I have enough information to help me deal…	46.80	
I am sure I have all the information I…	32.12	
I have all the information I need to…	24.17	
Range ****	22.63	
3. Actively managing my health	32.00	2.68 ± 0.60
I spend quite a lot of time actively managing…	35.00	
I make plans for what I need to do to be…	34.44	
Despite other things in my life. I make time…	22.50	
I set my own goals about health and fitness	31.11	
There are things that I do regularly…	35.75	
Range ****	13.25	
4. Social support for health	28.09	3.01 ± 0.55
I can get access to several people who…	48.88	
When I feel ill, the people around me really…	10.89	
If I need help. I have plenty of people I…	19.05	
I have at least one person…	23.46	
I have strong support from…	38.16	
Range ****	37.99	
5. Appraisal of health information	31.44	2.79 ± 0.55
I compare health information from different…	16.44	
When I see new information about health, I…	49.30	
I always compare health information…	25.63	
I know how to find out if the health…	25.14	
I ask healthcare providers about the quality…	40.67	
Range ****	32.86	
Part 2		(scored on a 1–5 scale)
6. Ability to actively engage with healthcare providers	54.94	3.28 ± 0.72
Make sure that healthcare providers understand…	70.59	
Feel able to discuss your health concerns with a…	64.99	
Have good discussions about your health…	45.66	
Discuss things with healthcare providers…	54.17	
Ask healthcare providers questions to get…	39.28	
Range ****	31.31	
7. Navigating the healthcare system	49.12	3.21 ± 0.69
Find the right healthcare	39.72	
Get to see the healthcare providers you need to	56.94	
Decide which healthcare provider you need…	64.72	
Make sure you find the right place to get…	38.06	
Find out what healthcare services you are…	46.67	
Work out what is the best care for you	48.61	
Range ****	26.66	
8. Ability to find good health information	51.40	3.46 ± 0.74
Find information about health problems	40.56	
Find health information from several…	57.10	
Get information about health so you are…	44.44	
Get health information in words you…	55.71	
Get health information by yourself	59.17	
Range ****	18.61	
9. Understand health information well enough to know what to do	39.93	3.64 ± 0.65
Confidently fill medical forms in the correct…	36.59	
Accurately follow the instructions from…	40.83	
Read and understand written health…	51.39	
Read and understand all the information on…	44.17	
Understand what healthcare providers are…	26.67	
Range ****	24.72	

* Items are truncated. Full items are available from the authors of the original HLQ. ** Proportions of responses indicating challenge for scales 1–5 were calculated as the proportions of “strongly disagree” and “disagree” responses versus “agree” or “strongly agree” responses. Proportions for scales 6–9 were calculated as the proportions of “cannot do or always difficult”, “usually difficult” and “sometimes difficult” responses versus “usually easy” and “always easy” responses. *** M—mean; SD—standard deviation. **** Range (min–max) of proportions of responses indicating challenge across a scale.

**Table 3:** Cronbach’s α and composite reliability (CR) values of the nine HLQ scales together with 95% confidence intervals.

Scale	1	2	3	4	5	6	7	8	9
Cronbach’s α (95% CI)	0.88 (0.85–0.91)	0.84 (0.80–0.87)	0.85 (0.82–0.88)	0.80 (0.75–0.83)	0.78 (0.74–0.82)	0.86 (0.83–0.88)	0.86 (0.83–0.88)	0.86 (0.83–0.89)	0.79 (0.75–0.83)
CR (95% CI)	0.87 (0.82–0.90)	0.85 (0.81–0.87)	0.85 (0.81–0.88)	0.79 (0.75–0.83)	0.76 (0.71–0.80)	0.85 (0.82–0.88)	0.85 (0.82–0.88)	0.86 (0.82–0.88)	0.79 (0.74–0.83)

CR—composite reliability; CI—confidence interval.

**Table 4:** Factor loadings of the HLQ items.

Scale Item Number	Factor Loadings in Part 1					Factor Loadings in Part 2			
	Scale 1	Scale 2	Scale 3	Scale 4	Scale 5	Scale 6	Scale 7	Scale 8	Scale 9
1. Feeling understood and supported by healthcare providers									
1.2	0.68								
1.8	0.80								
1.17	0.94								
1.22	0.82								
2. Having sufficient information to manage my health									
1.1		0.67							
1.10		0.68							
1.14		0.83							
1.23		0.83							
3. Actively managing my health									
1.6			0.76						
1.9			0.78						
1.13			0.68						
1.18			0.73						
1.21			0.71						
4. Social support for health									
1.3				0.66					
1.5				0.65					
1.11				0.77					
1.15				0.55					
1.19				0.67					
5. Appraisal of health information									
1.4					0.60				
1.7					0.59				
1.12					0.73				
1.16					0.65				
1.20					0.63				
6. Ability to actively engage with healthcare providers									
2.2						0.78			
2.4						0.69			
2.7						0.76			
2.15						0.75			
2.20						0.70			
7. Navigating the healthcare system									
2.1							0.64		
2.8							0.76		
2.11							0.70		
2.13							0.71		
2.16							0.70		
2.19							0.74		
8. Ability to find good health information									
2.3								0.74	
2.6								0.68	
2.10								0.82	
2.14								0.72	
2.18								0.73	
9. Understand health information well enough to know what to do									
2.5									0.63
2.9									0.56
2.12									0.70
2.17									0.69
2.21									0.71

**Table 5:** Factor correlations of the nine factors of the HLQ together with 95% confidence intervals (in brackets).

	F1	F2	F3	F4	F5	F6	F7	F8
HLQ Part 1 (scales 1–5)								
F2	0.34 (0.20–0.47)							
F3	0.26 (0.13–0.40)	0.68 (0.57–0.80)						
F4	0.57 (0.45–0.68)	0.43 (0.28–0.58)	0.43 (0.30–0.56)					
F5	0.35 (0.21–0.48)	0.75 (0.66–0.83)	0.75 (0.65–0.85)	0.57 (0.42–0.72)				
HLQ Part 2 (scales 6–9)								
F6	0.61 (0.52–0.71)	0.50 (0.39–0.61)	0.46 (0.35–0.58)	0.61 (0.50–0.71)	0.57 (0.47–0.67)			
F7	0.55 (0.44–0.65)	0.57 (0.46–0.67)	0.46 (0.35–0.57)	0.62 (0.53–0.71)	0.56 (0.46–0.67)	0.93 (0.89–0.97)		
F8	0.33 (0.20–0.47)	0.67 (0.58–0.76)	0.55 (0.44–0.66)	0.48 (0.33–0.62)	0.73 (0.64–0.82)	0.73 (0.63–0.83)	0.81 (0.73–0.89)	
F9	0.33 (0.20–0.47)	0.59 (0.49–0.68)	0.41 (0.29–0.53)	0.49 (0.36–0.62)	0.56 (0.45–0.68)	0.74 (0.65–0.83)	0.78 (0.70–0.86)	0.89 (0.82–0.96)

Each factor (F) represents a scale: F1. Feeling understood and supported by healthcare providers; F2. Having sufficient information to manage my health; F3. Actively managing my health; F4. Social support for health; F5. Appraisal of health information; F6. Ability to actively engage with healthcare providers; F7. Navigating the healthcare system; F8. Ability to find good health information; F9. Understand health information well enough to know what to do.

## Data Availability

The data supporting the findings of this study are available from the corresponding author upon reasonable request. Requests will be considered in accordance with ethical approval requirements and applicable institutional policies.

## Supplementary Materials

The following supporting information can be downloaded at: https://www.mdpi.com/article/10.3390/healthcare14193320/s1, Table S1. Factor loadings and 95% confidence intervals (CI) for the Health Literacy Questionnaire items, Table S2. Assessment of discriminant validity using the heterotrait–monotrait ratio among the nine Health Literacy Questionnaire scales.

## Abbreviations

The following abbreviations are used in this manuscript:

CFA	Confirmatory factor analysis
CFI	Comparative fit index
CFI	Confidence interval
CR	Composite reliability
HLQ	Health Literacy Questionnaire
HTMT	Heterotrait–monotrait ratio of correlations
M	Mean
RMSEA	Root mean square error of approximation
SD	Standard deviation
SRMR	Standardised root mean residual
TLI	Tucker–Lewis index
WHO	World Health Organization
WLSMV	Weighted least squares mean and variance adjusted estimator
χ^2^	Chi-square test statistic
