# Supplementary material for: The Assessment of Structural Validity and Internal Consistency of the Polish Health Literacy Questionnaire (HLQ) in Primary Care Settings
Source: Healthcare (Basel). 2026 Oct 6;14(19):3320. doi: 10.3390/healthcare14193320 (PMC13665345; doi:10.3390/healthcare14193320)
Supplement: Supplementary file 1 [file healthcare-14-03320-s001.zip › healthcare-4525242-supplementary.pdf]

Supplementary Material

Table S1. Factor loadings and 95% confidence intervals (CI) for the Health Literacy Questionnaire items.

Item number	Factor loadings in Part 1 (95% CI)					Factor loadings in Part 2 (95% CI)			
	**Scale 1**	**Scale 2**	**Scale 3**	**Scale 4**	**Scale 5**	**Scale 6**	**Scale 7**	**Scale 8**	**Scale 9**
Scale 1. Feeling understood and supported by healthcare providers									
1.2	0.68 (0.59-0.77)								
1.8	0.80 (0.72-0.87)								
1.17	0.94 (0.86-1.01)								
1.22	0.82 (0.71-0.92)								
Scale 2. Having sufficient information to manage my health									
1.1		0.67 (0.57-0.77)							
1.10		0.68 (0.59-0.78)							
1.14		0.83 (0.77-0.88)							
1.23		0.83 (0.78-0.89)							
Scale 3. Actively managing my health									
1.6			0.76 (0.67-0.84)						
1.9			0.78 (0.71-0.85)						
1.13			0.68 (0.60-0.76)						
1.18			0.73 (0.66-0.81)						
1.21			0.71 (0.63-0.79)						
Scale 4. Social support for health									
1.3				0.66 (0.56-0.76)					
1.5				0.65 (0.55-0.76)					
1.11				0.77 (0.70-0.84)					
1.15				0.55 (0.44-0.66)					
1.19				0.67 (0.59-0.76)					
Scale 5. Appraisal of health information									
1.4					0.60 (0.50-0.69)				
1.7					0.59 (0.49-0.69)				
1.12					0.73 (0.66-0.80)				
1.16					0.65 (0.56-0.75)				
1.20					0.63 (0.53-0.73)				
Scale 6. Ability to actively engage with healthcare providers									
2.2						0.78 (0.70-0.85)			

2.4						0.69 (0.62-0.75)			
2.7						0.76 (0.69-0.83)			
2.15						0.75 (0.70-0.81)			
2.20						0.70 (0.63-0.78)			
Scale 7. Navigating the healthcare system									
2.1							0.64 (0.57-0.72)		
2.8							0.76 (0.70-0.82)		
2.11							0.70 (0.64-0.77)		
2.13							0.71 (0.64-0.77)		
2.16							0.70 (0.62-0.77)		
2.19							0.74 (0.67-0.81)		
Scale 8. Ability to find good health information									
2.3								0.74 (0.68-0.81)	
2.6								0.68 (0.61-0.76)	
2.10								0.82 (0.76-0.87)	
2.14								0.72 (0.65-0.79)	
2.18								0.73 (0.66-0.80)	
Scale 9. Understand health information well enough to know what to do									
2.5									0.63 (0.54-0.71)
2.9									0.56 (0.45-0.66)
2.12									0.70 (0.63-0.77)
2.17									0.69 (0.61-0.76)
2.21									0.71 (0.63-0.79)

Table S2. Assessment of discriminant validity using the heterotrait-monotrait ratio among the nine Health Literacy Questionnaire scales.

	Scale 1	Scale 2	Scale 3	Scale 4	Scale 5	Scale 6	Scale 7	Scale 8	Scale 9
**Scale 1**	1.000								
**Scale 2**	0.334	1.000							
**Scale 3**	0.256	0.677	1.000						
**Scale 4**	0.554	0.430	0.422	1.000					
**Scale 5**	0.312	0.759	0.729	0.574	1.000				
**Scale 6**	0.603	0.496	0.458	0.600	0.556	1.000			
**Scale 7**	0.534	0.560	0.448	0.619	0.550	0.931	1.000		
**Scale 8**	0.299	0.683	0.543	0.478	0.729	0.722	0.804	1.000	
**Scale 9**	0.307	0.579	0.405	0.495	0.535	0.751	0.782	0.886	1.000
